# Epistemic austerity: limits to entitlement

**DOI:** 10.1007/s11229-021-03397-w

**Published:** 2021-09-14

**Authors:** Jakob Ohlhorst

**Affiliations:** University of Cologne, Cologne, Germany

**Keywords:** Epistemic entitlement, Hinge epistemology, Relativism, Deep disagreement, Problem of demarcation

## Abstract

Epistemic entitlement is a species of internalist warrant that can be had without any evidential support. Unfortunately, for this kind of warrant the so-called problem of demarcation arises, a form of epistemic relativism. I first present entitlement theory and examine what the problem of demarcation is exactly, rejecting that it is either based on bizarreness or disagreement in favour of the thesis that the problem of demarcation is based on epistemic arbitrariness. Second, I argue that arbitrariness generates a problem for entitlement because it undermines epistemic warrant. Third, I draw out some of the consequences that arbitrariness has for an entitlement epistemology, notably that it threatens to generalise to all our beliefs. Finally, I examine how different solutions to the problem of demarcation fare with respect to the danger of arbitrariness. I argue that none of the considered options succeeds in dealing with the risks of arbitrariness.

## Introduction

The idea that it is epistemically permissible to trust something to be true, without having evidence for it, has gained traction among internalists in recent years. They are inspired by Wittgenstein’s (1969) observation that we take certain propositions to be true, without having evidence speaking in their favour that is itself more warranted than these propositions. Nevertheless, we do not appear to be unreasonable in accepting these propositions. This debate has mostly played out under the label of *hinge epistemology*.

The idea is that these non-evidential convictions, which I will call hinges or hinge beliefs, can be epistemically warranted independently of evidence. Warrant comes instead from the special role that hinges play for our other beliefs because, without accepting non-evidential hinges, we would not be able to gain any evidence for other propositions and thereby any justification at all. We therefore are warranted to accept hinges by default. Wright (2004) calls this kind of warrant *epistemic entitlement*. Wright proposes several accounts of entitlement but in this paper I will focus on his account of *entitlement of cognitive project*.

On Wright’s view, we have two kinds of epistemic warrant: evidential justification for regular beliefs and entitlement to accept hinges which are a necessary presupposition for investigation. We need the latter kind, entitlement, in order to be able to get the more familiar evidential justification. Unfortunately, a problem arises for accounts of epistemic entitlement. Wright calls it the “problem of demarcation”, and he puts it as follows:

The point has not gone away that it is not in general, or even usually, consistent with responsible belief management to accept things without evidence or relevant cognitive achievement. What are the principles that determine when one may do so and when one ought not? How do we distinguish the genuine entitlements from the prejudices, mere assumptions and idées fixes? (Wright, 2014*, p. 245*)

The problem of demarcation asks the question how we can recognise actual cases of entitlement, and why some cases of hinges might not belong to this group. The questions I want to ask in this paper are: first, *what* exactly generates the problem of demarcation? Second, how is it *a problem*? As an example, consider a religious fundamentalist who has the hinge that the Bhagavad Gita is a reliable source of information. This fundamentalist will disagree with a materialist who has the hinge that current scientific research is veridical. Are both *equally entitled* to trust in these hinges?

At issue here is a special case of the debate over *relativism*: entitlement can be conceived of as strictly internal to the individual agent’s beliefs, unconstrained by any external influences. Thus, each individual agent’s entitlements function absolutely independently of any external considerations making each entitlement self-containing. Different individuals’ entitlements may be incompatible, but each one counts as equally warranted relative to her own position which leads to relativism. In this paper, I will develop an argument for why this relativism—why demarcation—is indeed a problem.

First, I will briefly introduce Wright’s view of epistemic entitlement. I will pay most attention to his account of entitlement of cognitive project, but I will also mention his other strategies. I then raise the problem of demarcation. Note, that Wright does not say much more about the problem than what I cited above. I will instead examine some ideas that might account for the problem: the bizarreness of some hinges as well as the phenomenon of deep disagreement. Neither approach gets at the heart of the issue, however. I will therefore argue for a third avenue: entitlements are epistemically arbitrary, and arbitrariness is problematic for entitlement. Finally, I will examine how the different solutions to the problem of demarcation that have been suggested until now fare under the arbitrariness reading of the problem of demarcation.

## Entitlement

Epistemic entitlement was developed as a response to the sceptical problem. Sceptical arguments aim to undermine our evidential justification for very fundamental beliefs. I call these beliefs hinge beliefs. For example, the beliefs that there are other minds or permanent physical objects are hinge beliefs. If we are not justified to accept these hinges, then all our other beliefs, which rely on these hinges, also lack justification. (Wright, 2004, p. 168).

Wright argues that, while we may lack evidential justification for our hinges, there may be some alternative kind of non-evidential warrant to be had for them. He calls this warrant epistemic entitlement because we can acquire it without any cognitive achievement. (Wright, 2004, p. 208).

Wright suggests several avenues for how we might be entitled to trust in these hinges. First, there is *strategic entitlement* which is based on Pascal’s Wager-style dominance reasonings and goes back to Reichenbach’s (1938) defence of induction. It weighs up what the best expected outcome is when we accept a hinge or remain agnostic about it. This account has received a fair amount of flak for its epistemic consequentialism with which I agree. (Pritchard, 2014; Elstein & Jenkins, 2020) I will therefore focus on another model of entitlement.

There are two accounts of entitlement that are less developed: entitlement of rational deliberation and entitlement of substance. The former argues that we need to accept hinges in order to act rationally; thus, we are entitled to accept them. The latter follows Strawson in a transcendental argument for entitlement from our conceptual schema to trust in the existence of the objects of experience. I will also bracket these two accounts.

Instead, I will focus on entitlement of cognitive project which takes epistemic activity, i.e. investigation, as the source of entitlement. This is the most Wittgensteinian account of entitlement which means that focusing on this argument may best help us to gauge the role of the problem of demarcation for hinge epistemology in general.

The idea here is that investigations necessarily come with a set of presuppositions^1^ which need to be true for a cognitive project to even make sense. To make an example: the cognitive project of weighing Henry the cat presupposes that my scale actually works. Now, I could take on the additional cognitive project of testing the accuracy of the scale, but this project would in turn engender further presuppositions and so on. As Wright puts it:

P is a presupposition of a particular cognitive project if to doubt P (in advance) would rationally commit one to doubting the significance or competence of the project. (Wright, 2004, p. 191)

We are entitled to accept presupposition P if the following two conditions hold additionally:

(i) We have no sufficient reason to believe that P is untrue

and

(ii) The attempt to justify P would involve further presuppositions in turn of no more secure a prior standing … and so on without limit; so that someone pursuing the relevant enquiry who accepted that there is nevertheless an onus to justify P would implicitly undertake a commitment to an infinite regress of justificatory projects, each concerned to vindicate the presuppositions of its predecessor. (Wright, 2004, p. 192)

These additional conditions restrict which kinds of presuppositions may be entitlements which brings us to the problem of demarcation. Some hinges will have to be disqualified from gaining entitlement status, but which and why? Are conditions (i) and (ii) enough? Apparently not, otherwise Wright would not have had to raise the problem of demarcation.

As an illustration, consider the ancient but still widely accepted hinge that the planets exert a causal influence on human affairs. We all live within the solar system and lack an “uninfluenced” control group. Consequently, we cannot get any defeating evidence against (i) or any non-circular supporting evidence for (ii) the principles of astrology. Note also that the other suggested accounts of entitlement do not posit any stricter restraints on entitlement; thus, the problem should arise for them equally. What does then undermine “responsible belief management” and pose the problem of demarcation?

## Bizarreness

There is a different passage in Wright’s (2004) that sounds a lot like the problem of demarcation:

More, there will be no obstacle in principle to the idea of alternative, equally valid ways of conceiving the substance of the world, either involving substitutions for our categories, or their augmentation in, as many would feel, bizarre and unmotivated ways. What are the barriers to an entitlement to wood spirits, ectoplasm, gods, and a plethora of existing but non-actual spatio-temporally unrelated concrete possible worlds? (Wright, 2004, p. 206)

Wright raises this issue only for the above-mentioned Strawsonian entitlement of substance. He introduced entitlement of substance because he did not think that the other strategies would be able to generate entitlement to trust in the existence of external objects and subjects. I am less sceptical about entitlement of cognitive project—namely I think that cognitive projects do indeed presuppose ontological hinge claims too and that we could not justify them with anything else than with an equally uncertain further hinge. Consider the cognitive project of figuring out what might have motivated Marie-Antoinette to her infamous saying; this project both presupposes a remote past and other minds, and if you felt committed to establish these hinges’ truth, then you would have to presuppose other hinges.^2^

Thus, the issue of bizarre ontologies may also arise for cognitive projects that are committed to such entities. As an example, consider modal investigations according to Lewis’s *On the Plurality of Worlds* (1986) where we presuppose the reality of possible worlds. Additionally, there may also be bizarre methods, for example astral projection. Consequently, the issue of bizarre hinges may generalise beyond entitlement of substance.

Are bizarre hinges the cause of the problem of demarcation? At least they are a case of it; if we want to solve the problem, then we also need to deal with bizarreness. In this section, I examine what bizarreness is and whether the problem of demarcation reduces to it.

If someone were to claim entitlement to believe in the existence of ectoplasm, then we would be extremely reluctant to grant it. If our theory of entitlement cannot block such cases, it would seem to be an epistemic free-for-all not an account of “responsible belief management”. But what is wrong with ectoplasm? Option 1 is that bizarreness means obvious falsehood which undermines entitlement. Option 2 is that bizarre views are so “unmotivated” that we could be entitled to just any (equally unmotivated) hinge.

The first option, equating bizarreness with obvious falsehood, would yield the following simple argument to explain the problem of demarcation. Assume for *reductio* that there are bizarre entitlements:

1. There are bizarre entitlements.
2. Bizarre beliefs are obviously false.
3. Entitlement is a form of warrant.
4. There is warrant for obvious falsehoods. (P1-3)
5. Something obviously false cannot be warranted.
6. Therefore, there are no bizarre entitlements. (C1, P4)

On this count, the problem of demarcation could be solved by excluding cognitive projects that take obviously false presuppositions on board. However, this clearly cannot be the issue of bizarreness given that Wright excludes presuppositions which we have sufficient reason to think that they are not true. Obvious falsehood clearly is a sufficient reason to think that a presupposition is not true; therefore, this is already excluded by Wright’s own account, and it cannot explain the problem of demarcation.^3^

Thus, what appears to be the problem of demarcation with bizarre hinges is Option 2, that they are unmotivated. If we can be entitled to accept something like *this* [a bizarre proposition], then we can be entitled to accept anything as true. I will come back to this thesis.

## Disagreement

Often beliefs are considered to be bizarre because we cannot understand how someone could even believe *this*. I think this lack of understanding hails from a divergence in hinges which shape profoundly different world views. This phenomenon has been called *deep disagreement*. It has enjoyed some discussion in social epistemology since at least (Fogelin, 1985).

Deep disagreement can be defined as disagreement about hinges. Given that we treat them as indubitable and beyond evidence, the only thing you can do in deep disagreement is recognising that the other disagrees while insisting that they are wrong. Does deep disagreement give us an avenue to come to grips with the problem of demarcation?

According to *conciliationism* (Goldman & O’Connor, 2019, sect. 3.2), disagreement from epistemic peers^4^ about whether P reduces our warrant for P. Might this be what demarcates entitlements from mere hinges: we are entitled to accept P only if no peers disagree that not-P?

The argument for this would run as follows:

1. There is peer disagreement about hinges.
2. Peer disagreement reduces the degree of warrant one has. (Conciliationism)
3. The entitlement to hinges is a form of weak warrant.
4. Peer disagreement defeats entitlement.^5^

On this view, the problem of demarcation is easily solved. Entitlement is excluded for whatever our peers and epistemic authorities would effectively disagree about.^6^ Thus, we would not be entitled to trust presuppositions for cognitive projects about which our epistemic peers disagree.

Consider Anne, absolute sovereign in her land and therefore supreme justice. If Anne has the cognitive project of judging some legal case and she presupposes the hinge that she is queen by divine right for this purpose, then she could claim entitlement of cognitive project for that hinge. But if Sarah who knows as much about law and theology as Anne disagrees with her, then Anne is not entitled to accept her divine support because of Sarah’s peer disagreement.

Taking disagreement to demarcate entitlement from unwarranted hinges would include the bizarreness criterion. What is found to be bizarre is stuff that is also being deeply disagreed about. A belief is bizarre because it is bizarre that someone could believe something like this.

You might object that, like obvious falsehood, this is already resolved by Wright’s own criterion that there cannot be a sufficient reason that speaks against the presupposition. That is, peer disagreement would not account for the problem of demarcation because Wright already excluded it.

Deep disagreement however, even by peers, does not appear to deliver a sufficient reason speaking against your hinges. Many authors writing about deep disagreement argue that we remain rational even if we stick to our guns in the face of peer disagreement about our hinges. (Hazlett, 2014; Ranalli, 2020) This arguably means that our warrant is not changed by the disagreement and that deep disagreement does not defeat entitlement.

I think this is the right result because not all deep disagreements are problematic or irresponsible. Take for example the divergent interpretations of quantum mechanics which arguably boil down to deep disagreements about the fundamental nature of reality; I doubt that either Bohr or Schrödinger lacked warrant for their hinges just because they disagreed with each other.

As a further point in case, if deep disagreement determined the problem of demarcation, entitlement would simply depend on your social context. To illustrate this: if Anne were queen with absolute power, then she could *create* the entitlement for her hinge that she is queen by divine grace. She would simply have to disappear Sarah and other peers who disagree about her divine grace. This would generate epistemic warrant by means that should clearly not be available. The only thing that this may generate is an appearance of epistemic entitlement.

Michael Lynch (2012, pp. 55–57) defends a more sophisticated version of the disagreement view. His reason to worry about the problem of demarcation and relativism is grounded in a view that deep disagreement is a fundamental problem for democratic or civil societies. If there is no shared epistemic fundament, a society is doomed to insuperable factionalism because no fundamental principles can be agreed upon. This motivation is distinctly pragmatist; agreement, rather than truth, is the target value. An entitlement would obtain if everyone agreed on a false hinge but not if a minority trusted a true hinge. I will examine Lynch’s proposal more closely later.

Nevertheless, I think that deep disagreement can illustrate what is going on in the problem of demarcation because deep disagreement also raises the question. Both disagreeing agents recognise that only one of them can be right. But each takes herself to be the one—and their hinges make them internally rational in taking themselves to be right and inferring the other’s wrongness from this.

Why does deep disagreement then illustrate the problem of demarcation? Because there can also be an outside perspective. By this, I do not mean the objective god’s eye view that an externalist advocates. Rather, it is what an uncommitted third agent, call her Abigail, would make of the two disagreeing agents’ positions. That is, I introduce a further neutral internal perspective, and on this intersubjective level deep disagreement becomes a problem.

By uncommitted I mean that Abigail has no interests or previous commitments and beliefs that make her prefer either of the disagreeing positions; she has no strong theological or political views that have any implications concerning queens’ divine grace in general or Anne’s in particular. Essentially, she can look at the options as naked propositions; her world view does not depend on it.

So, what would Abigail make of the deep disagreement about Anne’s divine grace, assuming that she has an interest in deciding this question? Arguably, she would be unable to commit to either side on basis of any reason. She would have to take a leap of faith in either direction because nothing favours either position. In other words, her choice will be epistemically *arbitrary*. She would be unable to tell why she picked the path she did at the moment of picking it. Note that Abigail’s predicament is in principle accessible to both Anne and Sarah.

## Arbitrariness

This arbitrariness, found with deep disagreement and a third party, extends to hinges in general. It generates the problem of demarcation. I do not claim that we are always confronted with Abigail’s choice when something becomes our hinge and entitlement—that is not how we acquire hinges—but Abigail’s situation represents our epistemic predicament.

We pick up hinges from our community and the practices we participate in. As a quite fine-grained example, consider the concept of marriage: while a society’s laws determine which relations count as marriages, different subgroups in the society will have different hinges about what marriage is *supposed to be*. Lawyers will just follow the legal practice, different religious groups will focus on their own religion’s precepts, and progressives think marriage is supposed to be highly inclusive. What someone considers to be a real marriage is in an important sense arbitrary and will mostly depend on how their community thinks about it. It is a mere contingency of our biography: we usually inherit our epistemic community’s entitlements.

A belief or action is adopted arbitrarily if and only if there is no non-circular reason that makes it preferable to alternative incompatible beliefs or actions. By extension, a belief is epistemically arbitrary if and only if there is no non-circular epistemic reason that makes it preferrable to incompatible beliefs. Martin Kusch calls this characteristic “non-neutrality”:

When judgements [attributing an epistemic status to a person or belief] (licensed by different standards of different frameworks) conflict, there are—at least in some important cases—no framework-independent, neutral ways of adjudication. (Kusch, 2019, p. 273)

I want to argue that arbitrariness is incompatible with warrant and *a fortiori* with entitlement. For this purpose, I can appeal to so-called underdetermination principles which take warrant to be undermined by the presence of equally warranted incompatible alternatives. We have only circular reasons and the entitling considerations speaking for entitlement—thus, they appear to be underdetermined because just about any hinge may have circular reasons and cognitive projects presupposing it. Your accepting one entitlement over the incompatible alternative is therefore arbitrary.

Vogel formulates the principle as follows: “Underdetermination Principle (UP): If q is a competitor to p, then one can know p only if one can *non-arbitrarily* reject q.” (Vogel, 2004, p. 427, my emphasis) Entitlement isn’t knowledge but a form of warrant, should this worry us? I don’t think so; Brueckner has a version for justification instead of knowledge. (Brueckner, 2005, p. 388) Also, Duncan Pritchard argues that underdetermination undermines the rationality part of rational knowledge. (Pritchard, 2016, p. 34) If underdetermination defeats rationality, arguably it also blocks warrant to trust. 

Usually, underdetermination is taken to undermine evidential justification. Does it extend to entitlement, i.e. non-evidential warrant? I believe that it does: if your non-evidential reasons underdetermine to which of two incompatible hinges you are more entitled, this entitlement would become arbitrary. Given the underdetermination, you could accept any alternative incompatible entitlement. Wright’s account would fail to demarcate entitlements from each other. If I cannot non-arbitrarily reject the alternative potential entitlements, then arguably I am not entitled to trust in my current hinge. Adopting such an arbitrary hinge would not be epistemically responsible.

This account also explains the issue of bizarreness following the second option that bizarre beliefs are unmotivated. If we cannot demarcate different hinges because they are all underdetermined, then we are in no way constrained from introducing other equally underdetermined hinges which may also be bizarre and unmotivated.

Thus, arbitrariness threatens entitlement in the following way:

1. Hinges are adopted arbitrarily without any evidential or non-evidential reasons favouring them over incompatible alternatives.
2. Arbitrariness is incompatible with epistemic warrant. (Underdetermination principle)
3. If hinges are adopted arbitrarily, then they are not warranted.

If this is the problem of demarcation, it may prove devastating to epistemic entitlement. Namely, the warrant-undermining arbitrariness risks to spread to all our beliefs because they rely on our hinges. This is an analogous issue to the so-called *leaching problem*. (Wright, 2004, p. 208) According to the leaching problem, we take up a risk in claiming an entitlement. All beliefs that rely on a risky entitlement will therefore inherit its risk, and they also inherit its diminished epistemic status. Essentially, all our beliefs will be no better off than our entitlements. They are as risky as the entitlement is.

If our hinges’ arbitrariness also leaches into all our beliefs, we can never be warranted in any belief. The demarcation problem in terms of arbitrariness threatens to force us into a global scepticism. It is therefore imperative for entitlement theory that it come to grips with the issue and solve the demarcation problem; otherwise, epistemic entitlement fails as an antisceptical strategy.

Is entitlement and entitlement of cognitive project in particular arbitrary? Note that (P1) conflated the hinges’ and the entitlements’ arbitrariness. Maybe the constraints that Wright posits get rid of the entitlements’ arbitrariness?

Every cognitive project comes with its presuppositions; if we are committed to the cognitive project, if we don’t have reason to doubt the project’s presuppositions, and if requiring reasons for the presuppositions would engender an infinite regress of further investigations, then we are entitled to trust the project’s presuppositions.

*Prima facie* we may then arbitrarily take up *any cognitive project*. Insofar, the cognitive projects are arbitrary. Wright however introduces some constraints on what projects may generate entitlement: first, the project’s presuppositions cannot be defeated by some reason we possess. This however is the *bare minimum*; it simply requires that we remain coherent. There are still plenty of cognitive projects that come with presuppositions compatible with our beliefs, i.e. the reasons we have, but which we would nevertheless consider to be utterly unmotivated and bizarre. Essentially, solving the problem of demarcation by requiring that our cognitive projects’ presupposition be undefeated does too little. As for the second constraint on entitlement from cognitive project: the regress condition does not effectively limit which cognitive project we may undertake because *all* cognitive projects come with presuppositions that would engender an infinite regress, even bizarre ones.

I therefore do not think that Wright’s criteria for entitlement of cognitive project can alleviate the problem of demarcation. They do not demarcate different cognitive projects, and thus they do not reduce the arbitrariness of the cognitive projects’ presuppositions. This underdetermination undermines the entitlement we were supposed to get from our cognitive projects.^7^

Another way to avoid the arbitrariness reading of the problem of demarcation would be to reject that the underdetermination principle applies to entitlement. The first way to do that is to reject it wholesale. I do not think that this is attractive. Given that the underdetermination principle raises the same issue as the problem of demarcation, rejecting underdetermination implies also denying that there is a demarcation problem.

The second way would be weakening the underdetermination principle: instead of defeating entitlement, arbitrariness only taints it. Thus, we would still be entitled, but underdetermined entitlement would be inferior to non-arbitrary entitlement. This would demarcate entitlement in a way, reducing the arbitrariness. However, I think this strategy is too timid. I take it that bizarreness shows that we do need to defeat inferior entitlements; otherwise, the view is too generous. Therefore, weakening the underdetermination principle should only be an anti-sceptical escape hatch if we do not succeed at solving the problem of demarcation in a satisfying manner.

My arbitrariness argument aligns with Duncan Pritchard’s argument for radical scepticism from underdetermination, (Pritchard, 2016, pp. 115–116) but I have to flag some differences.^8^ Notably, Pritchard’s argument aims at knowledge while I am only interested in the weak warrant that is entitlement. Also, he argues that underdetermination means that, for any humdrum belief, we fail to exclude the sceptical scenario. In the sceptical scenario we lack warrant, and radical scepticism ensues because we cannot exclude this bad case.

My arbitrariness view, meanwhile, being predicated on entitlement, is more convoluted and does not necessarily involve the sceptical scenario: given the few constraints on cognitive projects, we may be entitled to just about any hinge.^9^ We can claim entitlement for many incompatible hinges, making it arbitrary. This underdetermination of which presupposition we may pick and which we shouldn’t threatens our entitlement. Thus, my primary concern is the demarcation problem, not a sceptical argument; but through leaching we may have a sceptical problem on our hand.

## Proposed solutions to the problem of demarcation

I know of three approaches to address the problem of demarcation. First, there is Michael Lynch’s *method game* where a community negotiates which hinges should gain entitlement status, i.e. non-evidential warrant, behind a veil of ignorance. Lynch proposes this as a solution to a version of the problem of demarcation. According to Lynch, the problem is rooted in disagreement. An epistemic community’s establishing agreement gives us a criterion demarcating warranted hinges from mere convictions. I believe this approach is unduly optimistic, assuming more homogeneity among different ways of life than there effectively is.

The method game consists in a group of people negotiating the epistemic rules or hinges for a fictional epistemic community.^10^ The key point is that the players of the method game would join the epistemic community after the game has been played although they will not know with which social function or status and which hinges will actually be true. Thus, the chosen hinges will have consequences for them, but they cannot tell which exactly. This veil of ignorance is supposed to assure that the epistemic rules will be fair. (Lynch, 2012, pp. 97–98).

Lynch argues that we would get the simple hinges that we may rely on our “natural” capacities—sight, memory, language competence etc. Already that may be in jeopardy, at least if we consider the rich sceptical and idealist traditions throughout history, from ancient Greece to India, or just everyday scepticism. Lynch’s project needs to rely on agreement of some community. As soon as it includes actual adherents of a non-realist tradition, little agreement may be left.

But even if we grant minimal agreement on hinges about our natural faculties; limiting ourselves to entitlement to simple natural capacities would still mean epistemically crippling ourselves especially given that these capacities are not maximally reliable. Consequently, this minimal consensus would be far from optimal.

For that reason, Lynch argues that more agreement is possible. He argues that the method game would naturally converge on principles that are “repeatable, adaptable, intersubjective, and transparent”. (Lynch, 2012, p. 102) Repeatability means that a method will produce stable results. Adaptability means that a method is applicable to diverse topics. Intersubjectivity and transparency mean the accessibility of a method to everyone.

These features, according to Lynch, will fall out of a method game because we will not know which position we would be having behind the veil of ignorance. It would therefore be in our interest to have maximally public (intersubjectivity and transparency) and versatile (repeatability and adaptability) tools. What underlies his view is that our deep disagreements are products of our social positions, and our beliefs serve to preserve our power. Recall Lynch’s pragmatism. If we screen out power relations through a veil of ignorance, then it would be in our interest to defend a sort of epistemic egalitarianism.

I fear that this is much too optimistic. I would assume that any participant in a method game would be acquainted with some sort of experts and recognise that they are useful to have. Expertise is not transparent and not very intersubjective, be it the expertise of a high priest or the expertise of a nuclear physicist. Thus, we would probably end up with principles that are not accessible to everyone or even a majority.

Also, the emphasis on repeatability and adaptability, I think, radically underestimates the prevalence of anti-rationalist movements within human society. There have been medieval Christian or Muslim occasionalists, ancient Chinese legalists, modern defenders of revelation. Why would a veil of ignorance force one to abandon such disregard for reason? As mentioned, the method game needs to generate actual agreement among real participants.

More generally, I would wager that actual method games would not yield stable results.^11^ Agents would hardly be able to play the game “correctly”. Even if they managed to follow the rules, outcomes would vary widely given all sorts of psychological dispositions and biases.

This raises the question: how arbitrary is the outcome of the method game? The method game is a process *intended* to make a hinge epistemically less arbitrary through participant agreement but is the process any good for that purpose?^12^ I fear that the method game’s outcome will be highly contingent on who its participants are. I think it is no less arbitrary than the received wisdom of *any* society, and I do not think that either “it is received wisdom” or “we pragmatically negotiated these hinges” are reasons to demarcate one belief from the other.^13^

Second, there is Jochen Briesen’s (2012) development of entitlement of cognitive project. Briesen gives an account of cognitive projects as attempting to answer questions. Questions have a semantic structure that includes what the specific question *presupposes*. The question: “where was Siddhartha Gautama born?” presupposes that the person Siddhartha Gautama actually exists. If he does not, then the question has failed.

According to Briesen, we are entitled to accept the presupposition P of a cognitive project if and only if P is a presupposition of a *rational* and *promising* cognitive project to which we are committed, there have been no *preceding successful cognitive projects establishing the falsehood of P*, there are no defeaters against P, and P would otherwise be circularly justified. (Briesen, 2012, p. 251).

Briesen takes these criteria to avoid relativism and crazy epistemic presuppositions. I will read this as his approach to deal with the problem of demarcation. He takes the issue to be of a twofold nature: we cannot just adopt any arbitrary cognitive project because it could be internally contradictory or because it presupposes things contradictory to what we have already learnt. Thus, Briesen’s view attempts to solve the problem of demarcation through purely subject-internal restrictions.

His approach shifts the problem of demarcation onto the cognitive projects that we pursue instead of the hinges we adopt. The requirements of *rationality* and *promisingness* of projects are supposed to block relativism. Not just any arbitrary cognitive project can generate entitlements. The problem is that rationality and promisingness are too weak criteria.

A cognitive project is rational if and only if its presuppositions are consistent, and it is promising if and only if its presuppositions do not per se exclude the possibility to answer at least part of the question. There are plenty of abstruse cognitive projects that are consistent. Briesen only requires that the question does not presuppose a contradiction and that trying to answer the question is itself not hopeless. These two requirements do not yet reduce the arbitrariness of cognitive projects; the only thing they do is to exclude projects and entitlements that, *qua* inconsistency or irrationality, Wright or I would not even have considered as live options for entitlement.

The second requirement is stronger: if there have been preceding successful cognitive projects whose outcomes falsify the presupposition of some new cognitive project, then the new project is no live option for entitlement of cognitive project. This requirement, I believe, is too strong.

A first problem is that it might not model how actual cognitive projects work. In scientific practice measurements are frequently dismissed as measuring errors as they do not fit in with current theory. Consequently, not all outcomes of a particular cognitive project are weighted equally: some are ignored because they do not match with the presuppositions of the very project. There is some leeway as to what counts as a result of a cognitive project, and theory often takes the precedent over measurements. This kind of leeway is not possible if *all* outcomes of preceding cognitive projects prohibit changing our hinges. We require a certain flexibility in what counts as established, but this flexibility just reintroduces the problem of demarcation.

Second and more importantly, the outcomes of cognitive projects, although apparently successful, are sometimes false; we make mistakes. But if we are strictly bound by our precedent cognitive projects’ outcomes, we will be unable to correct past mistakes and to fundamentally revise or reject any theory. Essentially, the second criterion gets us stuck in whatever we currently think we know. It blocks any kind of theory change.

Consequently, the second requirement makes the outcomes of preceding cognitive projects indubitable, thus solidifying their arbitrariness. It reduces arbitrariness only relatively to preceding cognitive projects, thereby yielding a coherentist picture of entitlement. This coherentist structure of entitlement and justification, in its totality, may still be as arbitrary as ever. Also, the remaining criteria, the no-defeater condition as well as the non-circularity requirement, are beholden to whatever hinges we took for given in preceding cognitive projects. They do not avoid arbitrariness either.

The problem of Briesen’s solution to the problem of demarcation is that it is negative. While it accurately explains how entitlement gains its positive epistemic status, namely from the presupposition role it plays for investigation, it does not explain which questions are good enough to generate entitlement. Instead, it excludes certain questions as too bad as to generate entitlement. I argue that questions do not generate entitlement by default because we cannot avoid arbitrariness like that. Only *good* questions generate entitlement.

A third solution by Nikolaj Pedersen (2006) goes externalist. Pedersen points to the fact that entitlements per se are of epistemic value. Arguably, some are more valuable than others, so we can ask: which entitlements have more value? This is a way of posing the demarcation problem.

Pedersen’s solution is simple: true entitlements are obviously more valuable than false entitlements. So, true entitlements are *good*; false entitlements are *bad*. This makes some entitlements better than others and demarcates them. Note that this is an externalist solution. Good entitlements do not wear their truth on their sleeve. Rather, there is no way to figure out whether an entitlement is good or bad.

Indeed, I believe that the distinction between good and bad entitlement shows an important internal differentiation of entitlements: not all entitlements are created equal. This shows that even though there may be no means of rational change of hinges, we still may need to change them. However, I do not think that this distinction answers the problem of demarcation.

Given the arbitrariness analysis of the problem of demarcation, arbitrary hinges are not entitlements. Arbitrariness undermines entitlement. The problem of demarcation is not about merely false hinges; it is about dysfunctional hinges. Thus, Pedersen’s account solves one question: which entitlements are better than others? The problem of demarcation poses another question: which hinges are warranted and thereby entitlements? Arbitrariness will still guarantee that it is sheer luck if an entitlement happens to be good.

Second, even if it were used to solve the problem of demarcation, it would be very unsatisfying. Pedersen’s criterion does nothing to alleviate the arbitrariness of hinges because it is externalist. By stating that I am certain of some proposition and claiming entitlement to it, I cannot appeal to its goodness i.e. truth. Thus, whether the entitlement is good or bad changes nothing about its arbitrariness. Consequently, Pedersen’s account does not address the problem of demarcation.

## Conclusion

This paper set out to answer two questions: first, *what* exactly generates the problem of demarcation? Second, how is it *a problem*? I have argued that the problem of demarcation is a problem of arbitrariness which is incompatible with epistemic warrant because arbitrary states underdetermine entitlement.

First, entitlement is too unconstrained and therefore epistemically arbitrary. We need to demarcate some hinges from the arbitrary hinges to solve the problem demarcation and generate non-arbitrary entitlement for some hinges. Second, I have argued that epistemic arbitrariness undermines epistemic warrant which includes entitlement. Given the leaching problem, arbitrariness would spread to all our beliefs, leading to a new scepticism.

The problems of demarcation and arbitrariness also apply to relativism. A relativism that takes non-neutrality as its starting point has arbitrariness built in from the start. Relativists have several avenues available to deal with the arising demarcation problem: first, they could deny the underdetermination principle and embrace arbitrariness. This would however invite the full range of issues that the problem of demarcation produces: unmotivated and bizarre beliefs could multiply; an unrestricted propagation of world views would ensue. In sum, it would exacerbate the characteristics of relativism that make relativism unattractive to most philosophers.

Second, relativists might extend their relativism about epistemic status to a semantic relativism. Relativism about epistemic status would imply that justification is relative to our cultural context and our hinges. Semantic relativism would make truth relative to our cultural context and our hinges. By also endorsing semantic relativism, the relativist would virtually guarantee that relativist justification tracks relativist truth given that they are both sensitive to the same parameters. The problem of demarcation would not arise because entitlement and justification would all be truth-tracking. I think that such an approach profoundly misunderstands the function of epistemic warrant because it functionally collapses truth and warrant into one.^14^

Third, the relativist can recognise that she has a problem of demarcation and that even relativism needs to avoid or reduce arbitrariness. That is, even a relativist should recognise that not all cognitive projects and hinges are created equal and that we are only entitled to some of them. Hinge epistemology and epistemic entitlement have a strong relativistic bent as deep disagreement shows. I advocate for such a mitigated relativism.

I believe that the problem of demarcation can be resolved and that it is illustrative to look to ethics for this purpose. In ethics we aim at the good or right action just as we aim at truth in epistemology. Hinges and entitlement also aim at truth otherwise entitlement of cognitive project^15^ would seem deeply misguided. But just as it isn’t self-evident what the good or right is in ethics, it isn’t self-evident which hinges are true. (Wright, 2004, pp. 210–211).

One way in ethics is to go hard consequentialist, arguing that, independently of what we know, the best outcome determines the right action. This corresponds to Pedersen’s (2006) proposed solution of good and bad entitlement. I argued that that wasn’t satisfying as an account of epistemic entitlement.

Another solution in ethics is to require a good will to act right just as we try to trust the right hinges. This however leaves arbitrariness untouched. Even if we are motivated to do the right thing and to believe the truth, we arbitrarily follow the moral and epistemic prejudices that we happen to have. We might as well follow others and be equally motivated to try to discover truth and do the right thing. Our arbitrary presuppositions may lead us both morally and epistemically astray.

James Montmarquet (1992) proposed a solution to these issues jointly bedevilling ethics and epistemology: instead of focusing on single hinges and cognitive projects, we look at the agents. According to Montmarquet, we are required to be epistemically virtuous. More precisely Montmarquet demands the virtue of epistemic conscientiousness: our epistemic agency and character needs to be structured and informed by a deep care about the truth. It is not enough to just want to get things right. One also needs to enact and manifest this motivation. This care for the truth needs to inform our intellectual dispositions in order to produce a virtue.

I believe that intellectual virtue produces the “responsible belief management” (Wright, 2004, p. 204) that eliminates arbitrariness and solves the problem of demarcation. The argument here is that only cognitive projects that have been undertaken out of intellectual virtue will generate entitlement. Only hinges that effectively manifest a love of truth generate entitlement.

This demarcates virtuous from non-virtuous hinges and thereby reduces arbitrariness: given that some cognitive projects and their presuppositions will have been taken up virtuously but not others, it will not be underdetermined which presuppositions we are to trust. This also explains why Bohr and Schrödinger each may remain entitled in the face of disagreement and thereby an appearance of arbitrariness: they both adopted their incompatible hinges out of epistemic virtue. Allan Hazlett (2014, p. 7) similarly proposes that we can only claim entitlement if we have been virtuous or at least avoided epistemically vicious dispositions. I think that epistemic virtue and vice are the factor that demarcates arbitrary hinges from non-arbitrary or virtuous entitlements.

## Footnotes

1 Wright takes care to distinguish these presuppositions from hinge beliefs. However, one of the functions of hinges is to enable cognitive projects. (Wright, 2004, p. 190).

2 If you are really attached to the Strawsonian entitlement of substance, you might also argue that the cognitive project has a conceptual schema attached that entitles you to accept the existence of other minds and a remote past.

3 Also, if you are a fan of entitlement of substance, obvious falsehood should give you a reason not to accept that hinge.

4 A peer is someone who has the same evidence and competences as the subject.

5 This is one of the Pyrrhonian tropes. (Empiricus, 2000, p. 41).

6 On a more radical version, we could set the problem to what we *could* disagree about; however, that would open the gates wide for radical scepticism again. For that reason and because it looks like a fairly vacuous exercise to consider epistemic positions that no one actually would take, I shall ignore that option.

7 This arbitrariness extends to the other proposed types of entitlement: strategic entitlement allows almost any arbitrary epistemic gamble that isn’t self-defeating. Every hinge has a chance of getting it right. Also entitlements of rational deliberation or of substance do not indicate which hinges we should trust over others which is particularly visible in the issues of bizarreness and the deep disagreements we may have in rational deliberation.

8 I will bracket Pritchard’s (2016, p. 80) direct criticism of entitlement that trust is the wrong kind of state for hinges.

9 Indeed, we might argue that there are also sceptical cognitive projects sneaking the sceptical scenario right back in.

10 Lynch’s proposal is a series of votes – this may however lead to a Condorcet paradox, i.e. the game establishing inconsistent hinges. I therefore broadened this to some form of negotiation.

11 This is an empirical hypothesis.

12 This is the point where my diagnosis that the problem of demarcation boils down to arbitrariness rather than disagreement comes apart from Lynch’s. Agreement does not reduce arbitrariness, and non-arbitrariness does not force agreement.

13 Probably, long-term epistemic social processes are functionally equivalent to the evaluations of a method game. If that is true, then method games may turn out in all the different ways that epistemic communities have formed.

14 A variant of this strategy is hinge epistemology’s denial that hinges are either true or false.

15 As well as strategic entitlement.

## References

[CR1] Briesen, J. (2012). *Skeptische Paradoxa*. Mentis.

[CR2] Brueckner A (2005). Fallibilism, underdetermination, and skepticism. Philosophy and Phenomenological Research, LXX.

[CR3] Elstein, D., & Jenkins, C. I. (2020). The truth fairy and the indirect epistemic consequentialist. In N. Pedersen & P. J. Graham (Eds.), *Epistemic Entitlement* (pp. 344–360). Oxford University Press.

[CR4] Empiricus, S. (2000). *Outlines of Scepticism*. J. Annas and J. Barnes (Eds.). Cambridge University Press.

[CR5] Fogelin R (1985). The logic of deep disagreements. Informal Logic.

[CR6] Goldman, A. I. and O’Connor, C. (2019). Social Epistemology. In E. N. Zalta (Ed.) *The Stanford Encyclopedia of Philosophy* (Fall 2019). Retrieved September 1, 2021, from https://plato.stanford.edu/archives/fall2019/entries/epistemology-social/

[CR7] Hazlett A (2014). Entitlement and mutually recognized reasonable disagreement. Episteme.

[CR8] Kusch M (2019). II—Relativist stances, virtues and vices. Aristotelian Society Supplementary.

[CR9] Lewis, D. (1986). *On the plurality of worlds*. Wiley Blackwell.

[CR10] Lynch MP (2012). In praise of reason.

[CR11] Montmarquet J (1992). Epistemic virtue and doxastic responsibillity. American Philosophical Quarterly.

[CR12] Pedersen N (2006). Entitlements, good and bad. Nordic Journal of Philosophy.

[CR15] Pritchard, D. (2014). Entitlement and the groundlessness of our believing. In D. Dodd & E. Zardini (Eds.), *Scepticism and Perceptual Justification* (pp. 190–212). Oxford University Press.

[CR16] Pritchard D (2016). Epistemic Angst.

[CR17] Ranalli C (2020). Deep disagreement and hinge epistemology. Synthese.

[CR18] Reichenbach H (1938). Experience and prediction.

[CR19] Vogel J (2004). Skeptical arguments. Philosophical Issues.

[CR20] Wittgenstein, L. (1969). *Über Gewissheit - On Certainty*. G. E. M. Anscombe & G. H. von Wright (Eds.). Blackwell.

[CR21] Wright C (2004). Warrant for nothing (and foundations for free)?. Aristotelian Society Supplementary.

[CR22] Wright, C. (2014). On epistemic entitlement (II) - welfare state epistemology. In D. Dodd & E. Zardini (Eds.), *Scepticism and Perceptual Justification* (pp. 213–247). Oxford University Press.

